# Elevated Serum Levels of Interferon-Regulated Chemokines Are Biomarkers for Active Human Systemic Lupus Erythematosus

**DOI:** 10.1371/journal.pmed.0030491

**Published:** 2006-12-19

**Authors:** Jason W Bauer, Emily C Baechler, Michelle Petri, Franak M Batliwalla, Dianna Crawford, Ward A Ortmann, Karl J Espe, Wentian Li, Dhavalkumar D Patel, Peter K Gregersen, Timothy W Behrens

**Affiliations:** 1 Center for Immunology, University of Minnesota Medical School, Minneapolis, Minnesota, United States of America; 2 Department of Medicine, Johns Hopkins University School of Medicine, Baltimore, Maryland, United States of America; 3 North Shore Long Island Jewish Research Institute, Manhasset, New York, United States of America; 4 Thurston Arthritis Research Center, University of North Carolina at Chapel Hill, Chapel Hill, North Carolina, United States of America; University of Iowa, United States of America

## Abstract

**Background:**

Systemic lupus erythematosus (SLE) is a serious systemic autoimmune disorder that affects multiple organ systems and is characterized by unpredictable flares of disease. Recent evidence indicates a role for type I interferon (IFN) in SLE pathogenesis; however, the downstream effects of IFN pathway activation are not well understood. Here we test the hypothesis that type I IFN-regulated proteins are present in the serum of SLE patients and correlate with disease activity.

**Methods and Findings:**

We performed a comprehensive survey of the serologic proteome in human SLE and identified dysregulated levels of 30 cytokines, chemokines, growth factors, and soluble receptors. Particularly striking was the highly coordinated up-regulation of 12 inflammatory and/or homeostatic chemokines, molecules that direct the movement of leukocytes in the body. Most of the identified chemokines were inducible by type I IFN, and their levels correlated strongly with clinical and laboratory measures of disease activity.

**Conclusions:**

These data suggest that severely disrupted chemokine gradients may contribute to the systemic autoimmunity observed in human SLE. Furthermore, the levels of serum chemokines may serve as convenient biomarkers for disease activity in lupus.

## Abstract 2

A comprehensive survey of the serologic proteome in human SLE suggests that severely disrupted chemokine gradients may contribute to the systemic autoimmunity observed.

## Editors' Summary

**Background.:**

The term “lupus,” meaning wolf in Latin, is often used as an abbreviation for the disease systemic lupus erythematosus (SLE). The name may have been given because some people with SLE have a rash that slightly resembles a wolf's face. The condition affects around 50 to 100 people per 100,000, and is much more common in women than men. SLE is a complicated disease that comes about when antibodies inappropriately attack the body's own connective tissues, although it is not known why this happens. Symptoms vary between different people; the disease may get better and then worse, without explanation; and can affect many different organs including the skin, joints, kidneys, blood cells, and brain and nervous system. SLE is difficult for doctors to diagnose. Although the disease cannot be cured, patients who are diagnosed with SLE can be treated for their symptoms, and the right management can slow progress of the disease. One area of SLE research focuses on finding “molecular markers” (e.g., proteins or other compounds) that could be tested for in the blood. Researchers hope this would help doctors to more accurately diagnose SLE initially, and then also help to track progress in a patient's condition.

**Why Was This Study Done?:**

“Gene expression” is a term meaning the process by which a gene's DNA sequence is converted into the structures and functions of a cell. These investigators had found in previous studies that certain genes were more “highly expressed” in the blood cells of patients with SLE. Some of these genes were already known to be regulated by interferons (a group of proteins, produced by certain blood cells, that are important in helping to defend against viral infections). The investigators performing this study wanted to understand more clearly the role of interferon in SLE and to see whether the genes that are more highly expressed in patients with SLE go on to produce higher levels of protein, which might then provide useful markers for monitoring the condition.

**What Did the Researchers Do and Find?:**

This research project was a “case-control” study, in which the researchers compared the levels of certain proteins in the blood of people who had SLE with the levels in people who did not have the condition. Thirty people were recruited as cases, from a group of patients with SLE who have been under evaluation at Johns Hopkins School of Medicine since 1987. Fifteen controls were recruited from a group of healthy people of similar age and sex as the patients with SLE; everyone involved in the study gave their consent to take part. Blood samples were taken from each individual, and the serum (liquid component of blood) was separated out. The serum levels of 160 different blood proteins were then measured. When comparing levels of blood proteins between the groups, the researchers found that 30 specific proteins were present at higher or lower levels in the SLE-affected patients. Many of these proteins are cytokines, which are regulated by interferons and are involved in the process of “signaling” within the immune system. A few proteins were found at lower levels. Levels of the interferon-regulated proteins were, on average, seen at higher levels in people whose condition was more severe.

**What Do These Findings Mean?:**

These results suggest that patients with SLE are likely to have a very different pattern of regulation of certain proteins within the blood, particularly the proteins involved in signaling within the immune system. The authors propose that these proteins may be involved in the progression of the disease. There is also the possibility that some of these proteins may prove useful in diagnostic tests, or in tests for monitoring how the disease progresses. However, before any such tests could be used in clinical practice, they would need to be further developed and then thoroughly tested in clinical trials.

**Additional Information.:**

Please access these Web sites via the online version of this summary at http://dx.doi.org/10.1371/journal.pmed.0030491

- Patient information from the UK National Health Service on systemic lupus erythematosus
- Patient handout from the US National Institutes of Health
- MedlinePLUS encyclopedia entry on lupus
- Information on lupus from the UK Arthritis Research Campaign

## Introduction

Systemic lupus erythematosus (SLE, also called lupus) is a chronic, inflammatory autoimmune disease characterized by nuclear autoantibodies, immune complex formation, and systemic vasculitis [1]. Organs targeted in SLE include the skin, joints, lungs, blood cells, kidneys, and the central and peripheral nervous systems. The disease affects 0.1% of the population and shows a striking 9:1 female predominance. The factors contributing to the onset and progression of SLE are not well understood; however, genetic, environmental, and hormonal influences clearly contribute. SLE is difficult to diagnose, and the disease course is characterized by unpredictable flares and remissions. Thus, there is a pressing need to develop biomarkers that will allow for more accurate diagnoses and facilitate better assessment of disease activity and organ involvement.

Recently, we, and others, have used gene expression microarrays to discover a group of interferon (IFN)-regulated mRNA transcripts that are expressed at elevated levels in the blood cells of most SLE patients [2–4]. The IFNs are a family of cytokines originally identified on the basis of their ability to mediate cellular resistance to viral infection [5]. They influence cell proliferation, differentiation, and survival of mature lymphocytes, class switching at immunoglobulin heavy chain loci and activation of dendritic cells (DCs), and have a central role in host defense against infections and tumorigenesis [6]. The type I IFNs consist of 13 IFN-α subtypes, IFN-β, and IFN-Ω, which act through the same two-subunit receptor (IFNAR1 and IFNAR2) [6]. Type I IFNs are induced by viruses, bacteria, and synthetic compounds such as double-stranded RNA and imiquimod, and are produced mainly by plasmacytoid DCs and macrophages. Type II IFN, IFN-γ, acts through distinct receptor subunits (IFNGR1 and IFNGR2) and is produced by T, natural killer, and natural killer T cells in response to T cell receptor stimulation and cytokines.

In addition to the recent gene array findings in SLE [2–4], there is a growing body of evidence linking type I IFN and lupus pathogenesis. About 20% of human patients treated with IFN-α developed positive blood tests for anti-nuclear antibodies, and there are case reports of individuals treated with IFN-α going on to develop SLE (reviewed in [7]). Elevated levels of serum IFN-α are detectable in human SLE patients [8], and these levels are sufficient to induce the maturation of normal monocytes into activated DCs [9]. Type I IFN pathway activation in SLE may result, in part, from the triggering of intracellular toll-like receptors (TLR7 and TLR9) expressed in plasmacytoid DCs by nucleic acid-containing immune complexes [7]. Genetic variation in toll-like receptor signaling pathways, such as recently shown for IRF5 [10], is also likely to be important for SLE pathogenesis. The ability of IFN-α to stimulate differentiation of B cells to antibody-secreting plasma cells [11] suggests the possibility of a feed-forward cycle contributing to disease. Here we report the results of a comprehensive analysis of serum proteins in SLE.

## Methods

### Research Participants, Samples, and Clinical Data

SLE patients were enrolled from the Hopkins Lupus Cohort [12] under the auspices of the National Institutes of Health/National Institute of Arthritis and Musculoskeletal and Skin Diseases-Sponsored Autoimmune Biomarkers Collaborative Network, following informed consent. Healthy age- and gender-matched controls were recruited at the University of Minnesota (Minneapolis, Minnesota, United States). Blood was collected by peripheral venipuncture and the sera separated in serum-separator Vacutainer tubes (Becton-Dickinson, Palo Alto, California, United States). A protease inhibitor (aprotinin, 1 μg/ml) was added to each sample, and aliquots were immediately frozen at −80 °C.

Whole blood gene expression profiles were obtained on blood collected concurrently using the PaxGene system (Qiagen, Valencia, California, United States/Becton-Dickinson, Franklin Lakes, New Jersey, United States). cRNA probes were generated from PaxGene RNA and hybridized with Affymetrix U133A chips (Santa Clara, California, United States) using standard protocols. The levels of 82 IFN-regulated genes that distinguish SLE from controls were normalized and used to assign a gene expression “score,” as described [2]. Fifteen patients with the highest up-regulation of IFN-regulated transcripts (IFN-hi) and 15 patients with lower levels of the same transcripts (IFN-lo) were studied, in addition to 15 healthy controls selected randomly from a larger collection of age- and gender-matched controls.

Detailed clinical data were available for each visit and included the disease activity measures SLE Disease Activity Index (SLEDAI) and Systemic Lupus Activity Measure, laboratory test results, and medication profiles. The SLEDAI [13] consists of 22 defined, weighted items grouped into nine organ systems. The index is calculated by summing all weighted items that were present within the previous ten days. Possible SLEDAI scores range from 0 to 101. The Systemic Lupus Activity Measure-Revised (SLAM-R) lists 33 clinical and laboratory manifestations of SLE [14]. Each manifestation is graded according to the severity of activity within the month before evaluation. Possible total scores range from 0 to 86.

### Serum Protein Measurements

The levels of 160 serum protein analytes were measured in serum aliquots (100 μl) from the cases and controls using custom dual-antibody sandwich immunoassay arrays, as described (Table S1) [15,16]. Briefly, monoclonal capture antibodies specific for each analyte were fixed to glass slides, with 12 replicate spots for each analyte. Duplicate samples of sera were incubated for 2 h and then washed. Slides were then incubated with secondary biotinylated polyclonal antibodies, and signals were amplified using a “rolling circle” method [16]. Quality control measures included optimization of antibody pairs, the use of internal controls to minimize array-to-array variation, and standardized procedures of chip manufacturing [15,16]. Arrays were scanned using a Tecan LS200 (Tecan, Männedorf, Switzerland) and mean fluorescence intensities were generated with customized software. To convert mean fluorescence intensities to concentration values, 15 serial dilutions of recombinant analytes at known concentrations were used to develop best-fit equations for each analyte. Four anchor-point control dilutions of recombinant analytes were included on each slide. The upper and lower limits of quantitation were defined to ensure a dynamic working range. An additional 12 analytes were measured on the protein microarrays (beyond the 160) for which 80% or more of the samples were at the upper or lower limits of detection; these were excluded from further analysis (Table S1).

In order to validate a subset of the results obtained from the microarray platform, we measured the levels of two of the chemokines, CCL2 (MCP-1) and CXCL9 (MIG), in 40 serum samples (15 IFN-hi, 12 IFN-lo, and 13 controls) using Biosource, Protein Multiplex Immunoassays (Invitogen, Carlsbad, California, United States) coupled with Luminex xMAP technology (Luminex, Austin, Texas, United States). Samples were run in duplicate, and calibrated recombinant proteins were used to generate standard curves. The coefficient of variance for duplicates averaged 10.7%. Linear regression was used to calculate correlation coefficients between the results obtained on the microarray platform and the Luminex assays after the data had been log_2_ transformed. These analyses indicated a high level of concordance between the two platforms (Figure S1). CXCL10 (IP-10) was not included on the antibody microarray panel, and levels were measured by Luminex assays.

### In Vitro Type I IFN Stimulations

Peripheral blood mononuclear cells (PBMCs) from healthy donors were isolated using lymphocyte separation medium (Mediatech Cellgro, Herndon, Virginia, United States). PBMCs were resuspended in complete medium (RPMI 1640 consisting of 2 mM L-glutamine, 50 units/ml penicillin, and 50 μg/ml streptomycin) + 10% autologous plasma at 2 million cells/ml. Cells were incubated in medium alone or with 1,000 units/ml each IFNα and IFNβ (R & D Systems, Minneapolis, Minnesota, United States) for 6 or 24 h, and total RNA was isolated and converted to cRNA (Ambion, Austin, Texas, United States), which was then hybridized to Affymetrix U133A gene expression arrays and analyzed using Microarray Suite 5.0. Transcripts were considered IFN-regulated if there was a mean change in expression of greater than 2-fold when compared to PBS control at either 6 or 24 h, together with a difference in expression of greater than 500 Affymetrix expression units as compared to control incubations.

### Statistical Methods

Clinical features of the IFN-hi and IFN-lo groups were compared using Student's unpaired t-test for continuous variables or the Fisher's exact test for binary data. The levels of individual analytes were compared between the IFN-hi, IFN-lo, and control sample groups using Student's unpaired t-test. Analyte levels and continuous clinical variables generally followed a normal distribution. To confirm the results generated by t-tests we used the nonparametric Mann-Whitney test (Table S1). Corrections for multiple testing were performed using the method of Benjamini and Hochberg (Table S1) [17]. Also, one-way ANOVA was used to confirm results from pairwise t-tests (Table S1). Concentration values for each analyte of interest were normalized to the mean of controls, log_2_ transformed, and then subjected to unsupervised hierarchical clustering (CLUSTER) and visualized using TreeView [18] (both programs can be downloaded free of charge at the Web site of Michael Eisen: http://rana.lbl.gov/EisenSoftware.htm).

The levels of seven IFN-regulated chemokines—CCL2 (MCP-1), CCL3 (MIP-1α), CCL8 (MCP-2), CCL19 (MIP-3β), CXCL9 (MIG), CXCL10 (IP-10), and CXCL11 (I-TAC)—were used to calculate a “chemokine score.” Concentration values were normalized across all samples so that the maximum value for any analyte was 1.0, and values for each sample were then summed to derive the final score.

Clinical data including disease activity indices (SLEDAI, SLAM-R) and laboratory results (anti-DNA antibodies, complement levels, erythrocyte sedimentation rates [ESR], white blood cell [WBC] counts, and hematocrit) were compared with analyte data using linear regression analysis (Pearson's correlation). One of the IFN-lo cases had insufficient clinical data and was excluded from the regression analyses. To determine the statistical significance of each comparison, random permutation analysis was performed to define the *p*-value thresholds.

To assess the possible role of chemokines in organ-specific disease involvement, we identified those patients who showed evidence for active disease as determined by the organ specific features of the Physician's Global Assessment, SLEDAI, and SLAM-R, or by laboratory testing. The patient group was then divided into two groups (positive and negative), and Student's unpaired t-tests were used to compare chemokine levels between the groups.

## Results

**Figure 1:**
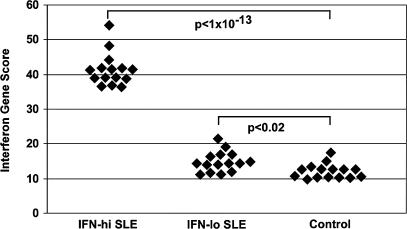
IFN Gene Expression Scores for IFN-hi SLE, IFN-lo SLE, and Control Participants Whole blood gene expression microarrays were used to identify 82 type I IFN-regulated genes that distinguished 81 SLE cases from 42 controls. From these genes, a normalized IFN gene score was derived. Plotted are IFN gene scores for 15 IFN-hi SLE (mean ± SD, 41.0 ± 4.8), 15 IFN-lo SLE (14.4 ± 2.8), and 15 controls (12.1 ± 1.9).

In an effort to build upon our recent gene expression studies of peripheral blood cells [2], we used a novel high-throughput protein microarray platform [15,16] to measure the levels of 160 analytes in blood serum of SLE cases and controls. Two groups of SLE cases were studied. The first demonstrated high levels of IFN-regulated mRNA transcripts (IFN-hi) in whole blood as determined by gene expression arrays, while the second group had low levels of these transcripts (IFN-lo), similar to those of controls (Figure 1).

Table 1 compares the clinical and demographic features of the two groups. The IFN-hi group was enriched for African-American women (8/15) compared with the IFN-lo group (2/15; *p* = 0.05) and, on average, fulfilled more criteria for SLE (7.3) than IFN-lo cases (5.7; *p* = 0.004). All of the IFN-hi cases had a history of positive anti-dsDNA antibodies and either low C3 or C4 complement levels, while these features were found in only about half of the IFN-lo group. The IFN-hi group also showed evidence of more active disease than the IFN-lo group as determined by higher SLEDAI and SLAM-R scores, together with several laboratory measures characteristic of active disease (low complement C3 and elevated ESR and anti-double-stranded DNA antibodies) at the time of the visit. There were no significant differences in medication profiles between the groups. Thus, similar to our earlier observations in an independent cohort of SLE cases [2], disease severity appears to be increased in IFN-hi as compared to IFN-lo patients.

**Table 1:**
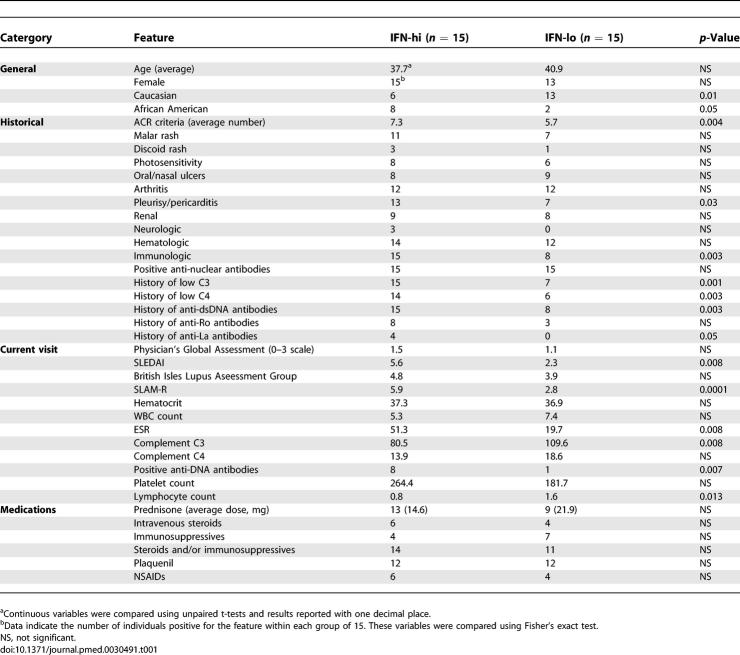
Clinical and Demographic Features of SLE Cases

**Figure 2:**
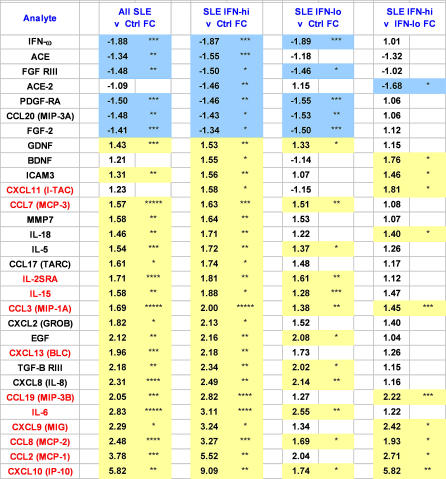
Thirty Protein Analytes Dysregulated in SLE Serum Sera from 15 IFN-hi SLE, 15 IFN-lo SLE, and 15 controls were assayed for the presence of 160 protein analytes by antibody microarrays. CXCL10 (IP-10) was measured by Luminex bead immunoassays. Shown are 30 analytes that demonstrated significant differences in at least one inter-group comparison, using the following criteria: mean fold change (FC) ≥ 1.5, and *p* < 0.05 by unpaired t-test. Data are presented as mean fold change of the group comparisons. Analytes regulated by type I IFN, as determined by in vitro gene expression microarray experiments, are highlighted in red font. * *p* < 0.05; ** *p* < 0.01; *** *p* < 0.001; **** *p* < 0.0001; ***** *p* < 0.00001. Comparisons highlighted in yellow represent significant positive associations, and blue indicates significant negative associations. The levels of statistical significance shown represent nominal *p*-values and are not corrected for the multiple hypotheses tested. The results obtained when these *p*-values were corrected using the Benjamini-Hochberg adjustment for multiple testing are provided in Table S1.

Of the 160 analytes measured, 30 (19%) showed significant differences in serum levels in at least one intergroup comparison (Figure 2). All 30 analytes were different between the IFN-hi SLE group and controls, and most varied between IFN-lo SLE and controls. Surprisingly, expression levels for most of the analytes (19/30 [63%]) were not significantly different between IFN-hi and IFN-lo SLE. The majority of analytes identified (23/30 [77%]) exhibited higher serum levels in one or more of the SLE groups compared to controls. Notably, the levels of most CD4+ T helper-1 (TH1) cytokines (interleukin [IL]-2, TNF-α, and IFN-γ) and T helper-2 (TH2) cytokines (IL-4, IL-9, IL-10, and IL-13) were similar in both SLE and control groups (Table S1).

**Figure 3:**
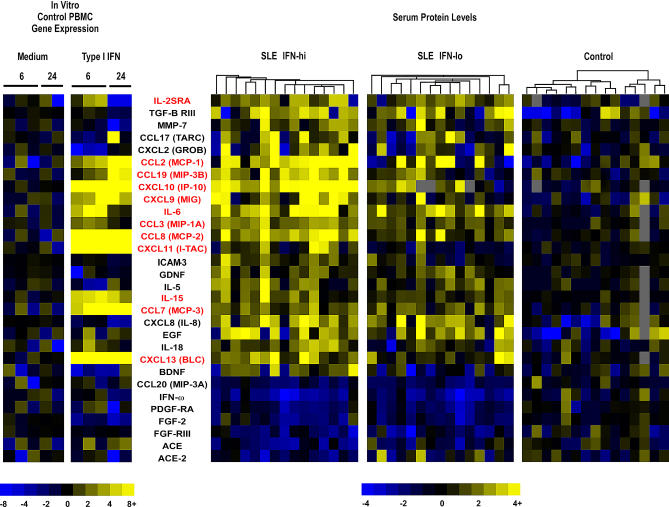
Coordinate Dysregulation of Serum Protein Levels in SLE Hierarchical clustering was applied to protein levels of the identified analytes (“Serum Protein Levels”). Individual data points represent the log_2_ ratio of the analyte concentration to the mean of control concentrations (scale shows linear fold-differences). “In Vitro Control PBMC Gene Expression” columns present gene expression microarray data obtained by incubating normal control PBMCs in vitro with medium alone or with type I IFN for 6 and 24 h. Data are normalized to control medium-alone conditions (scale depicts linear fold-differences). Grey boxes indicate missing data. Analytes induced by type I IFN in vitro (>2-fold and >500 expression unit mean difference) are highlighted in red font.

Down-regulated analytes included IFN-Ω, angiotensin converting enzyme, angiotensin converting enzyme-2, the chemotactic cytokine (chemokine) CCL20 (MIP-3A), the growth factor FGF-2, and soluble growth factor receptors (FGF-R3, PDGF-RA). Up-regulated analytes included several cytokines (IL-5, IL-6, IL-15, IL-18, BDNF, and GDNF), the cytokine receptors IL-2SRA and TGF-B RIII, matrix metalloproteinase 7, and the adhesion molecule ICAM-3. Strikingly, 12 of the 23 up-regulated analytes identified were chemokines and included representatives of both the CC- and CXC- families [19]. Chemokines are an important group of soluble mediators that function to recruit leukocytes to inflammatory sites and guide the homeostatic movement of immune cells to specific subregions of secondary lymphoid tissues [20]. Most of the chemokines identified are inducible by type I IFN (highlighted in red in Figure 2, and see Figure 3 below). Seven of the 11 analytes that differed in serum levels between IFN-hi and IFN-lo SLE cases were IFN-regulated chemokines.

Hierarchical clustering analysis showed that many of these proteins were coordinately dysregulated in the serum of a large percentage of IFN-hi patients and in a smaller fraction of IFN-lo cases (Figure 3). The left-hand portion of Figure 3 show the results of 6 and 24 h stimulations of normal control PBMCs with type I IFN in vitro and identify analytes that were transcriptionally regulated by type I IFN as determined by gene expression arrays. From these data we conclude that many SLE patients are in an IFN-regulated chemokine “storm,” with broad dysregulation of serum protein profiles. Furthermore, the highest levels of IFN-inducible analytes are found in patients carrying the IFN blood cell gene signature.

**Figure 4:**
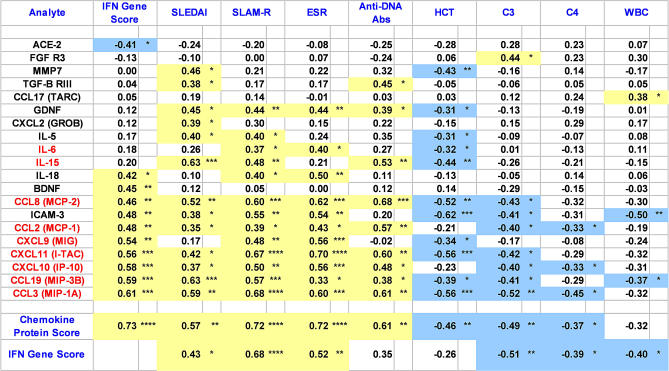
IFN-Regulated Chemokines Are Associated with SLE Disease Activity Shown are 20 serum analytes that exhibited significant positive (highlighted in yellow) or negative (highlighted in blue) correlation coefficients with clinical measures of SLE. The IFN gene score was calculated from 82 IFN-inducible transcripts measured by concurrent whole blood gene expression microarrays. The chemokine protein “score” was calculated using the seven IFN-regulated CC and CXC chemokines highlighted in red. * *p* < 0.05; ** *p* < 0.01; *** *p* < 0.001; **** *p* < 0.0001. *p*-Values were obtained by permutation testing.

For the majority of individual analytes, there was a very poor correlation between the levels of gene expression observed in blood and their protein levels in serum (Figure S2). In contrast, the IFN-regulated chemokine protein levels were generally highly correlated with the presence of IFN-responsive gene transcripts in blood (IFN gene “score”) (Figure 4). The discordance between chemokine protein and mRNA levels in blood may reflect the short half-life of chemokine and cytokine mRNAs or, alternatively, may indicate that the primary sites of chemokine production are extra-vascular. The latter seems likely, given that many chemokines are synthesized locally in tissues in response to inflammatory stimuli, transcytosed through vascular endothelial cells lining venules and capillaries [21], and then presented on the luminal surface of vessels for interaction with rolling leukocytes.

Twenty analytes showed one or more significant correlations with clinical measures of disease activity (Figure 4). Many of the IFN-regulated analytes showed strong positive correlations with the SLEDAI [13] and the SLAM-R [14], two validated measures of global disease activity, as well as with the ESR and titers of anti-dsDNA antibodies. The IFN-regulated analytes were negatively correlated with hematocrit and complement C3 levels and showed similar negative trends with complement C4 levels and WBC counts.

An IFN-regulated chemokine protein “score,” derived from the normalized levels of seven analytes, reflected the trends observed with the individual IFN-inducible analytes and showed stronger associations than any single analyte (Figure 4). The data also suggest that the chemokine score is more highly correlated with disease activity, as measured by SLEDAI, SLAM-R, ESR, and anti-DNA antibodies, than the IFN gene score for these individuals (Figure 4). We conclude that there are striking clinical correlations of many analytes, especially those that are IFN-regulated, with various measures of SLE disease activity.

We next asked whether the chemokine levels measured in serum were correlated with specific organ system involvement. We divided the patients on the basis of the presence or absence of organ system disease activity at the time of the visit (renal, serositis, hematologic, and skin) and then compared levels of individual chemokines (Tables 2 and S2). CXCL11 (I-TAC), CXCL13 (BLC), CXCL10 (IP-10), and CCL3 (MIP-1A) were present at significantly higher levels in serum of patients with active renal disease than those without. Levels of CCL8 (MCP-2), CCL2 (MCP-1), and CXCL2 (GROB) trended towards significance. The overall chemokine score was also elevated in patients with renal disease as compared to those without (Table 2).

**Table 2:**
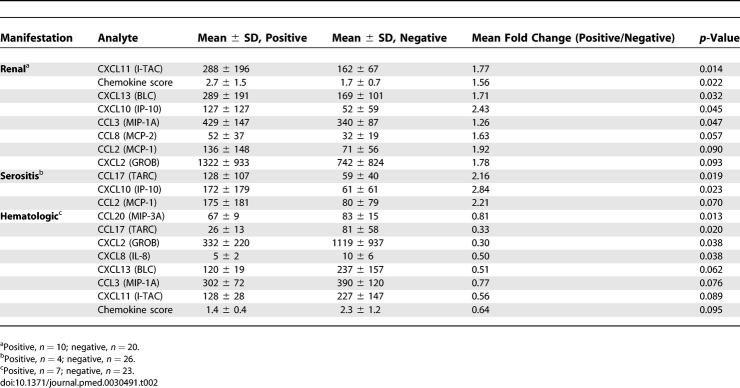
Chemokine Levels and Clinical Features of SLE

Higher levels of CCL17 (TARC), CXCL10 (IP-10), and CCL2 (MCP-1) were found in the small subset of patients (*n* = 4) with active serositis. Interestingly, a negative correlation was observed between levels of CCL20 (MIP-3A), CCL17 (TARC), CXCL2 (GROB), and CXCL8 (IL-8) and active hematologic system involvement (mostly thrombocytopenia) (Table 2), and several additional chemokines (CXCL13 [BLC], CCL3 [MIP-1α], and CXCL11 [I-TAC]) trended towards significance. No significant correlations were observed for patients with skin disease (Table S2). In a reciprocal approach, we ranked the 30 patients by serum levels of individual chemokines, divided each group evenly into chemokine “X” high and low groups, and compared clinical features (Figure S3). These data generally mirrored the findings shown in Table 2 and demonstrated that many chemokines were associated with nephritis in this sample, and that lower chemokine levels were generally observed in individuals with hematologic involvement.

## Discussion

Using a comprehensive protein microarray survey of the serologic proteome, we identified 30 dysregulated protein analytes in SLE, the majority of which were not previously implicated in systemic lupus. Furthermore, we demonstrate that SLE patients carrying the IFN gene signature are at particular risk for a dramatic coordinate dysregulation of IFN-responsive cytokines and chemokines in serum, and this pattern is strongly associated with various measures of disease activity. We hypothesize that high levels of systemic chemokines in active SLE, driven by type I IFN, lead to a state of “chemokine confusion” that alters the normal trafficking and chemotaxis of leukocytes in the body, setting the stage for widespread, systemic autoimmunity. High-level production of chemokines may also contribute to human SLE by recruiting immune and inflammatory cells into target tissues and by impairing the normal localization of certain populations of immune cells in the body, perhaps through chemokine receptor desensitization [22].

An interesting finding of this study is the similarity of cytokine/chemokine profiles between IFN-hi and IFN-lo SLE cases (Figures 2 and 3), including elevated levels of many IFN regulated analytes in IFN-lo serum. There are several potential explanations for these findings. First, IFN-lo patients may have stimuli other than type I IFN driving cytokine and chemokine expression, although we believe this to be unlikely. Second, the production of type I IFN in IFN-lo patients may be highly localized to tissues or secondary lymph organs, such that the gene expression “footprints” of interferon stimulation are not measurable in the blood of these patients. Third, IFN-lo patients may produce lower levels of type I interferon that are sufficient to induce IFN-responsive chemokines and cytokines, but insufficient to result in a strong IFN gene expression signature in blood cells. Regardless of the precise mechanism, the data suggest that type I interferon may be contributing to the pathogenesis of SLE in the majority of adult patients, as previously shown for pediatric SLE [3].

These data confirm previous studies reporting elevated levels of IL-6 [23], IL-15 [24], IL-18 [25], and the soluble IL-2 receptor alpha [26] in SLE serum. Interestingly, classically defined TH1 and TH2 cytokines such as IFN-γ, IL-2, IL-4, IL-5, and IL-10 did not show significant differences in serum levels between SLE and controls (see Table S1). Although many studies have explored the TH1/TH2 paradigm in SLE [27], there is no dominant consensus as to how TH1/TH2 pathways contribute to SLE. The lack of skewing of TH1/TH2 cytokines in the current study suggests either that this paradigm is not a defining feature of systemic lupus, or that measurement of these cytokines in serum is not a sensitive gauge of the status of these pathways in SLE.

Our conclusion that IFN-regulated chemokines are important in SLE disease pathogenesis is supported by a number of reports in the literature. Early studies suggested that serum and urine levels of CCL2 (MCP-1) [28,29] and CXCL8 (IL-8) [30,31] were associated with active lupus nephritis. A recent report by Rovin et al. [32] showed that CCL2 (MCP-1) levels in urine are a much better marker for flares of renal disease than CXCL8. Surface levels of CCR4, the receptor for CCL17 (TARC), are elevated on CD4+ T cells in active SLE [33], and lupus nephritis biopsies show high numbers of infiltrating CCR4+ T cells [34]. Other studies in SLE have described elevated serum levels of CCL5 (RANTES) [35], CXCL10 (IP-10) [36], the chemokine fractalkine [37], and CXCL9 (MIG) [38] in SLE, with levels often correlating with disease activity. Recent studies in cutaneous lupus erythematosus (CLE) also suggest an important role for chemokines and their receptors in mediating autoimmune skin inflammation. The chemokines CXCL9 (MIG), CXCL10 (IP-10), and CXCL11 (I-TAC) and the type I IFN-inducible intracellular protein MxA were found at elevated levels in skin lesions of CLE patients [39,40], and these sites were also enriched for CXCR3+ T cells [39,40], while the blood was relatively depleted [40]. CCL17 (TARC) and T cells bearing the CCR4 receptor were also found in the epidermis of CLE cases [41].

The current data demonstrate, to our knowledge for the first time, the coordinated up-regulation of IFN-regulated chemokines and cytokines in SLE and clearly link the expression of many of these analytes to the IFN gene signature. Together, these data suggest that the targeting of T cell-dependent autoimmune responses to various SLE end organs (e.g., kidney, skin, pleura, and central nervous system) may be mediated by local production of IFN-responsive chemokines. CXCL9 (MIG), CXCL10 (IP-10), and CXCL11 (I-TAC) were present at elevated levels in SLE serum and are known to recruit CXCR3+ effector and memory T cells, as well as natural killer cells and a subset of plasma cells to inflammatory sites. CCL2 (MCP-1), CCL3 (MIP-1α), CCL7, and CCL8 (MCP-2) are chemotactic for T cells and DCs, and may additionally contribute to systemic inflammation. The clinical observation that patients with active systemic lupus are often leukopenic may be related to chemokines driving lymphocytes out of the vascular space, as suggested for CXCR3+ T cells in CLE [40]. The negative correlations between WBC counts and IFN-regulated chemokine levels lend further support for this idea (Figure 4).

High serum and tissue levels of chemokines are known to desensitize immune cells to the normal chemotactic action of endothelial chemokines or tissue gradients [22]. In mice, systemic overexpression of MCP-1 impaired the ability of monocytes to home to a localized MCP-1 gradient [42]. This mechanism could explain the unusual presence of antibody secreting plasmablasts and plasma cells in the blood of SLE patients [43], a situation that is generally not observed in healthy individuals due to the normal homing of plasma cells along CXCL12 (SDF-1) gradients to the bone marrow [44]. Another possible consequence of the chemokine “storm” is impaired recruitment of Foxp3+ T regulatory cells to inflammatory sites. T regulatory cells suppress ongoing autoimmune responses, and their recruitment depends on interactions between CCR4 and its ligand CCL22 [45]. We show here that CCL17 (TARC), an alternate ligand for CCR4, is up-regulated in many SLE patients, and we speculate that this may lead, via receptor desensitization, to impaired chemotaxis of T regulatory cells into autoimmune inflammatory sites. Elevated levels of the homeostatic chemokine CXCL13 (BLC), which targets CXCR5+ B cells to follicles, and CCL19 (MIP-3β), which directs T cells, B cells, and DCs into the T cell zones of secondary lymph tissue, could also mediate ectopic lymphoid aggregates or germinal centers in nonlymphoid tissues [46]. Another potential result of elevated chemokine levels in SLE is heightened costimulatory signaling for T cells during cognate interactions with antigen-presenting cells [47]. Overall, the correlations between chemokine levels and organ system involvement observed in the current study are extremely complex (Tables 2 and S2), and additional work will be required with an increased sample size to understand how individual chemokines are contributing to specific organ inflammation in SLE.

Other analytes identified in this screen are also likely contributing to the widespread immune activation observed in SLE. Matrix metalloproteinase 7 has been shown to participate in the establishment of chemokine gradients at mucosal surfaces [48]. IL-6 is a major positive regulator of B cell differentiation into plasma cells, and IL-15 and sIL-2R function to control the proliferation of T cells. Major challenges for the future will be to determine how these soluble mediators synergize for the induction and maintenance of autoimmune responses in SLE, to further explore the relationship between the unique cytokine and chemokine milieu present in individual patients, and the profound clinical heterogeneity characteristic of SLE.

In conclusion, this study provides new perspective on the nature and extent of the immune dysregulation in lupus and lends further support for a central role of type I IFN in SLE pathogenesis. The data indicate that monitoring chemokine levels in blood of lupus patients may be useful clinically in assessing lupus disease activity. Finally, these findings suggest that new therapies aimed at blocking type I IFN or chemokine action may be effective in SLE.

## Supporting Information

Figure S1 Measurement of CCL2 (MCP-1) and CXCL9 (MIG) Using Luminex Bead-Based Immunoassays Linear regression analysis comparing the results obtained using the protein antibody microarrays and Luminex bead immunoassays for the analytes CCL2 (MCP-1; *r* = 0.91) and CXCL9 (MIG; *r* = 0.8) after the data were log_2_ transformed. Units are pg/ml.

Figure S2 Weak Correlation Between Serum Protein Levels and Blood Gene Expression Levels Linear regression analysis was used to measure the correlations of individual protein levels with concurrent gene expression levels in whole blood for the patient and control groups as determined by Affymetrix microarrays. Dotted lines indicate *p* < 0.05 thresholds. Analytes regulated by type I IFN are highlighted in red font.

Figure S3 Incidence of Organ System-Specific Disease Involvement in Patient Groups Divided by Serum Chemokine Levels For each chemokine, patients were segregated into two groups of 15 (chemokine “X” Hi, chemokine “X” Lo) based on serum chemokine concentrations (pg/ml). Bar graphs represent the incidence of lupus disease manifestations (skin, serositis, renal, and hematologic involvement) at the time of the visit. Fisher's exact test was used to determine *p*-values. * *p* < 0.05; ** *p* < 0.01.

Figure S4 Chemokine Protein Scores Chemokine scores were calculated as described in the Methods section and are plotted for 15 IFN-hi SLE (mean ± standard deviation [SD], 2.8 ± 1.2), 15 IFN-lo SLE (1.4 ± 0.5), and 15 controls (1.0 ± 0.2).

Table S1 Mean Serum Concentrations for 161 Analytes Mean serum analyte concentrations (pg/ml) for 15 IFN-hi SLE cases, 15 IFN-lo SLE cases, and 15 controls.

Table S2 Serum Chemokine Levels and Organ System Involvement Serum chemokine levels (pg/ml) and specific SLE organ system involvement at the time of the visit.

## Abbreviations

CLE: cutaneous lupus erythematosus
DC: dendritic cell
ESR: erythrocyte sedimentation rate
IFN: interferon
IL: interleukin
PBMC: peripheral blood mononuclear cell
SD: standard deviation
SLAM-R: Systemic Lupus Activity Measure-Revised
SLE: systemic lupus erythematosus
SLEDAI: SLE Disease Activity Index
TH: T helper
WBC: white blood cell
