# Supplementary material for: Elevated Serum Levels of Interferon-Regulated Chemokines Are Biomarkers for Active Human Systemic Lupus Erythematosus
Source: PLoS Med. 2006 Dec 19;3(12):e491. doi: 10.1371/journal.pmed.0030491 (PMC1702557; doi:10.1371/journal.pmed.0030491)
Supplement: Supplementary file 1 [file pmed.0030491.sg001.pdf]

FIGURE S1

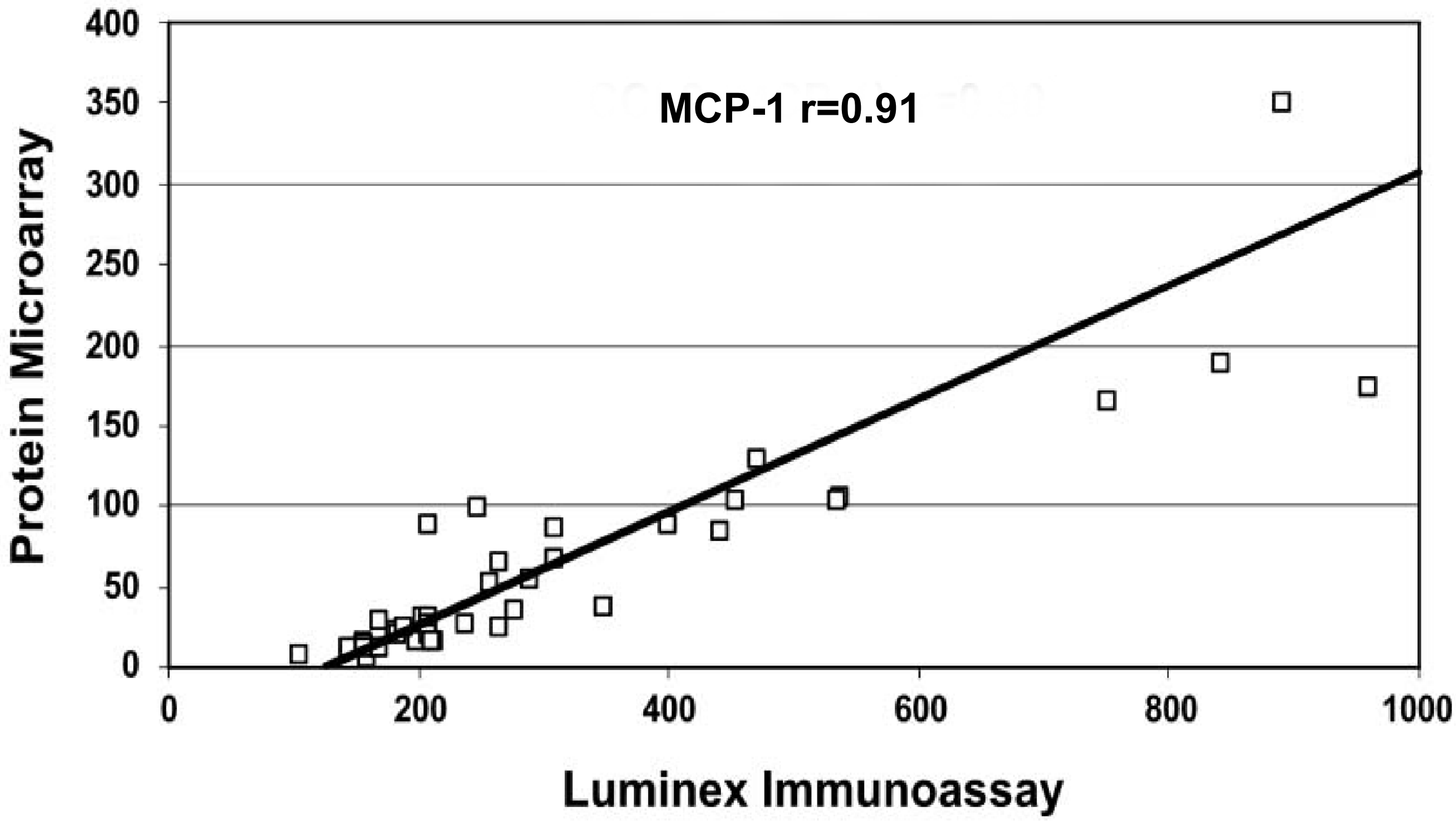
MCP-1 r=0.91
Protein Microarray
Luminex Immunoassay
0
50
100
150
200
250
300
350
400
0
200
400
600
800
1000

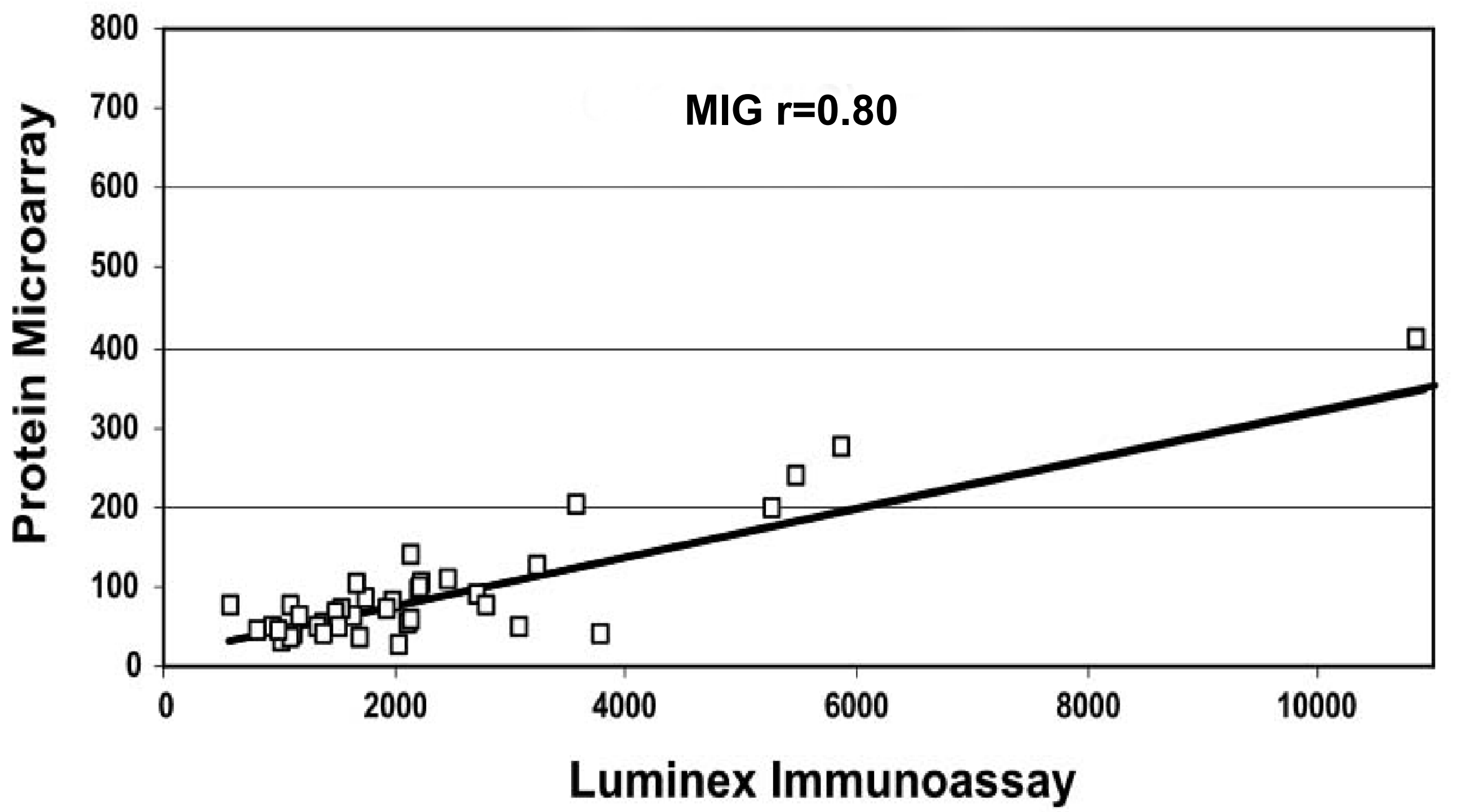
MIG r=0.80
Protein Microarray
Luminex Immunoassay
0
100
200
300
400
500
600
700
800
0
2000
4000
6000
8000
10000
