# Supplementary material for: Elevated Serum Levels of Interferon-Regulated Chemokines Are Biomarkers for Active Human Systemic Lupus Erythematosus
Source: PLoS Med. 2006 Dec 19;3(12):e491. doi: 10.1371/journal.pmed.0030491 (PMC1702557; doi:10.1371/journal.pmed.0030491)
Supplement: Supplementary file 2 [file pmed.0030491.sg002.pdf]

FIGURE S2

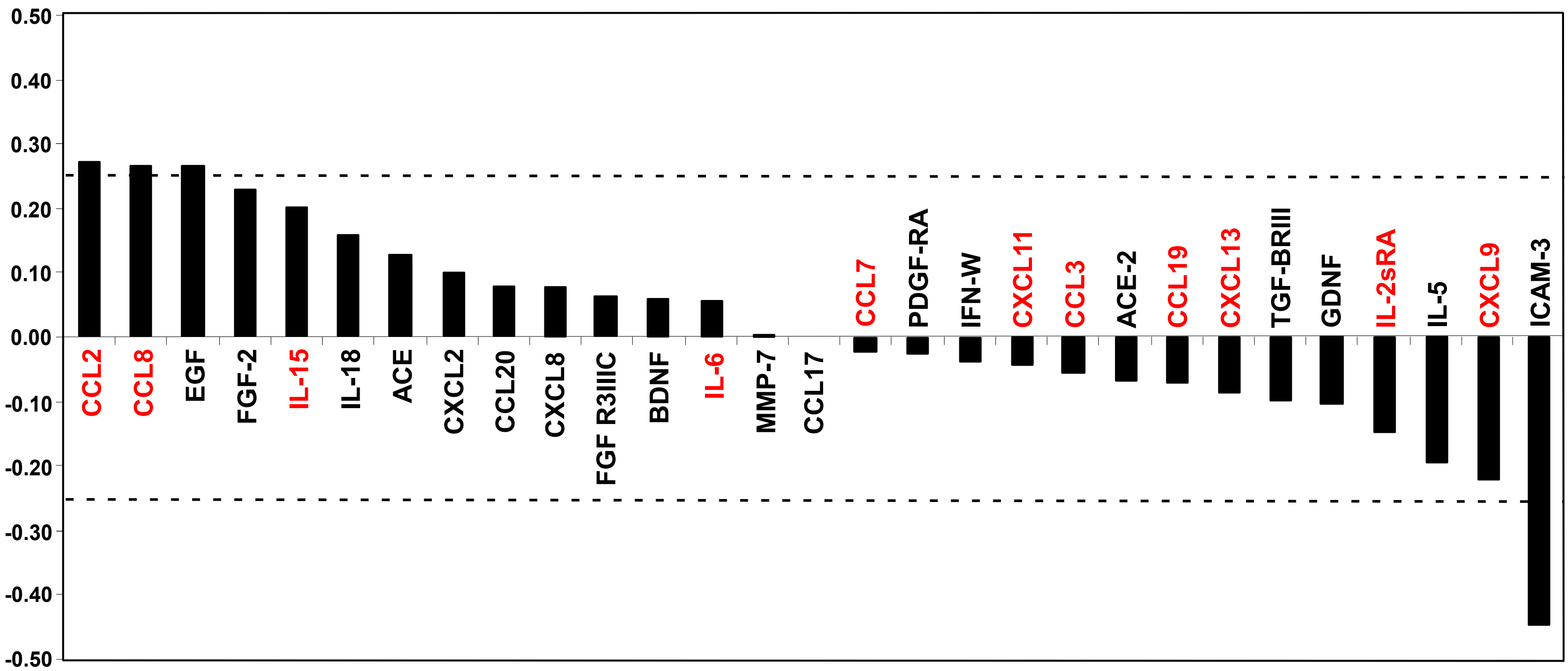

0.50
0.40
0.30
0.20
0.10
0.00
-0.10
-0.20
-0.30
-0.40
-0.50
CCL2
CCL8
EGF
FGF-2
IL-15
IL-18
ACE
CXCL2
CCL20
CXCL8
FGF R3IIIC
BDNF
IL-6
MMP-7
CCL17
CCL7
PDGF-RA
IFN-W
CXCL11
CCL3
ACE-2
CCL19
CXCL13
TGF-BRIII
GDNF
IL-2sRA
IL-5
CXCL9
ICAM-3
