# Supplementary material for: Elevated Serum Levels of Interferon-Regulated Chemokines Are Biomarkers for Active Human Systemic Lupus Erythematosus
Source: PLoS Med. 2006 Dec 19;3(12):e491. doi: 10.1371/journal.pmed.0030491 (PMC1702557; doi:10.1371/journal.pmed.0030491)
Supplement: Supplementary file 3 [file pmed.0030491.sg003.pdf]

FIGURE S3

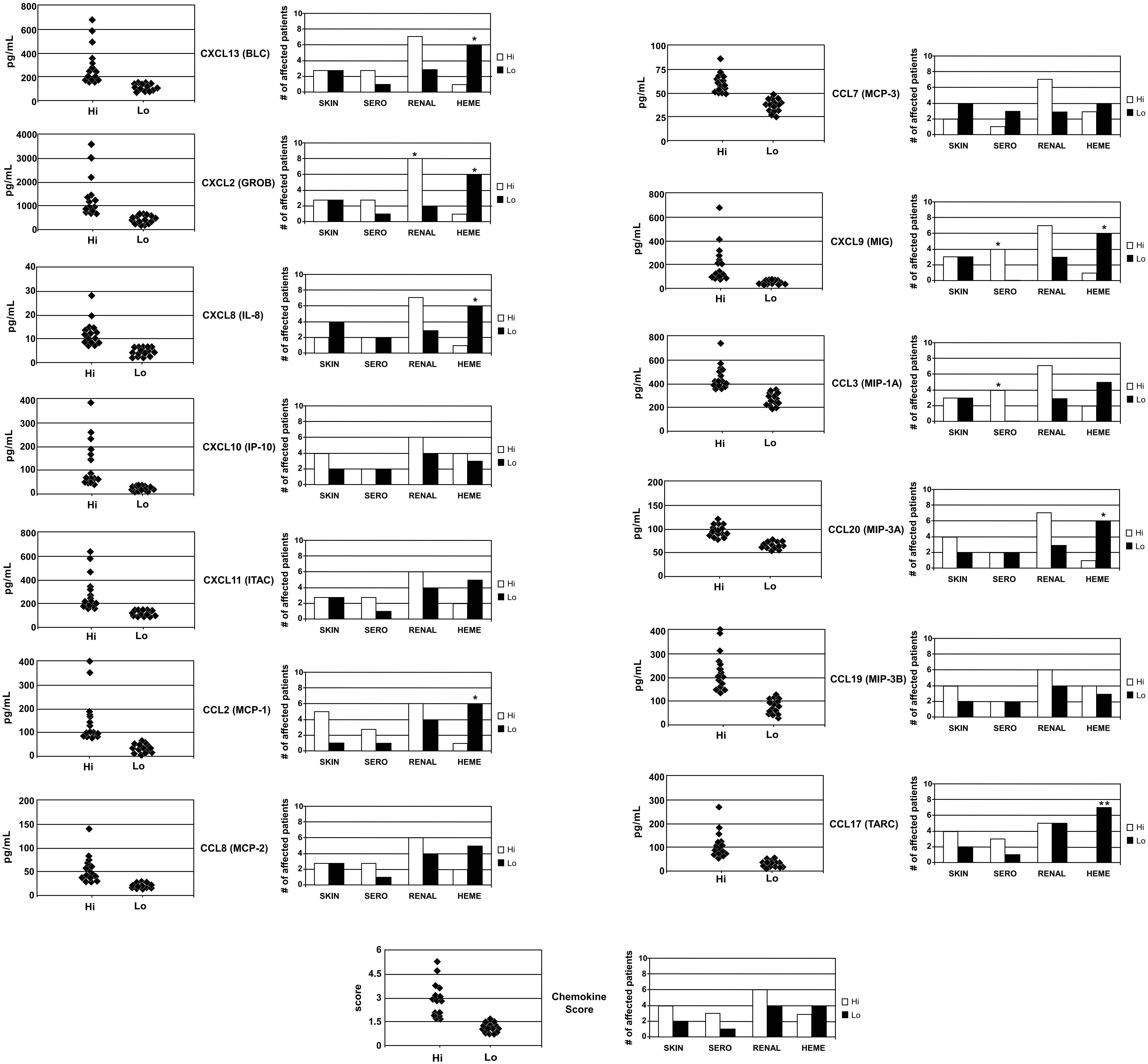

pg/mL
# of affected patients
Hi
Lo
SKIN
SERO
RENAL
HEME
CXCL13 (BLC)
CXCL2 (GROB)
CXCL8 (IL-8)
CXCL10 (IP-10)
CXCL11 (ITAC)
CCL2 (MCP-1)
CCL8 (MCP-2)
CCL7 (MCP-3)
CXCL9 (MIG)
CCL3 (MIP-1A)
CCL20 (MIP-3A)
CCL19 (MIP-3B)
CCL17 (TARC)
score
Chemokine Score
