# Supplementary material for: Elevated Serum Levels of Interferon-Regulated Chemokines Are Biomarkers for Active Human Systemic Lupus Erythematosus
Source: PLoS Med. 2006 Dec 19;3(12):e491. doi: 10.1371/journal.pmed.0030491 (PMC1702557; doi:10.1371/journal.pmed.0030491)
Supplement: Supplementary file 4 [file pmed.0030491.sg004.pdf]

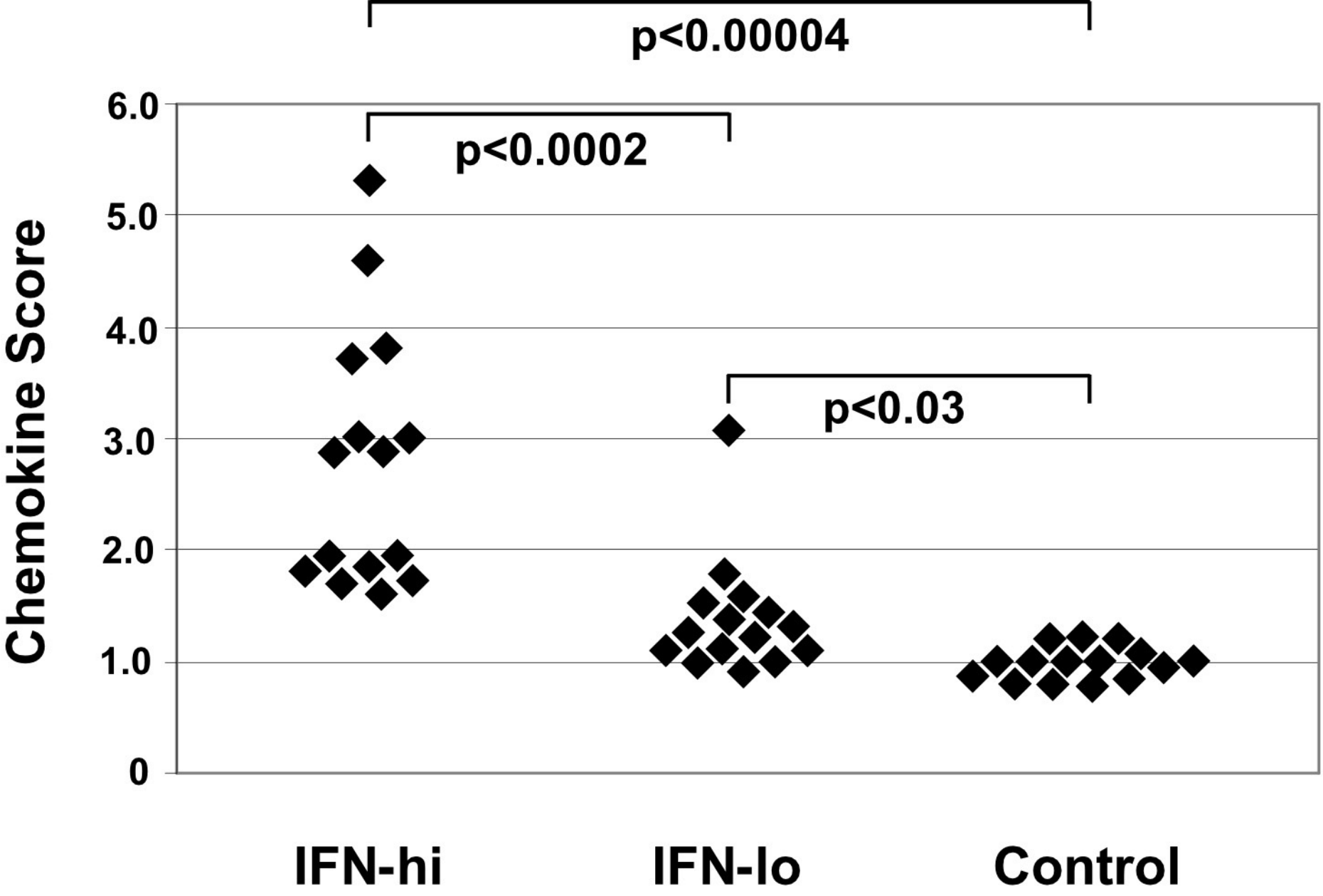
p<0.00004
p<0.0002
p<0.03
Chemokine Score
6.0
5.0
4.0
3.0
2.0
1.0
0
IFN-hi
IFN-lo
Control
